# Ischemic Preconditioning in the Liver Is Independent of Regulatory T Cell Activity

**DOI:** 10.1371/journal.pone.0049647

**Published:** 2012-11-21

**Authors:** Luke R. Devey, James A. Richards, Richard A. O’Connor, Gary Borthwick, Spike Clay, A. Forbes Howie, Stephen J. Wigmore, Stephen M. Anderton, Sarah E. M. Howie

**Affiliations:** Centre for Inflammation Research, The Queen’s Medical Research Institute, University of Edinburgh, Edinburgh, United Kingdom; CNRS, France

## Abstract

Ischemic preconditioning (IPC) protects organs from ischemia reperfusion injury (IRI) through unknown mechanisms. Effector T cell populations have been implicated in the pathogenesis of IRI, and T regulatory cells (Treg) have become a putative therapeutic target, with suggested involvement in IPC. We explored the role of Treg in hepatic IRI and IPC in detail. IPC significantly reduced injury following ischemia reperfusion insults. Treg were mobilized rapidly to the circulation and liver after IRI, but IPC did not further increase Treg numbers, nor was it associated with modulation of circulating pro-inflammatory chemokine or cytokine profiles. We used two techniques to deplete Treg from mice prior to IRI. Neither Treg depleted FoxP3.LuciDTR mice, nor wildtyoe mice depleted of Tregs with PC61, were more susceptible to IRI compared with controls. Despite successful enrichment of Treg in the liver, by adoptive transfer of both iTreg and nTreg or by in vivo expansion of Treg with IL-2/anti-IL-2 complexes, no protection against IRI was observed.We have explored the role of Treg in IRI and IPC using a variety of techniques to deplete and enrich them within both the liver and systemically. This work represents an important negative finding that Treg are not implicated in IPC and are unlikely to have translational potential in hepatic IRI.

## Introduction

Ischemia reperfusion injury (IRI) is the cause of considerable morbidity and mortality across a wide range of common diseases. In liver resection surgery, ischemic insults cause post-operative transaminasemia and failure of synthetic function. In liver transplantation, IRI causes a spectrum of graft dysfunction, ranging from biochemical abnormalities through to primary non-function of the transplanted organ [1].

Ischemic preconditioning (IPC) is a manoeuvre in which brief interruption, then reinstatement of an organ’s blood supply protects that organ from subsequent injury. IPC has been shown to protect the liver from subsequent IRI in animals and humans [2], [3]; its mechanism of action has yet to be fully elucidated.

In recent years, IRI has been thought of as an example of sterile inflammation, and the contribution of lymphocytes has been examined extensively. A transient T cell influx has been described, occurring during the first few hours of reperfusion, and resolving by 24 hours [4], [5]. Nude [6], [7], RAG1 knockout [8], SCID [9], TCRαβ−/− [10], and CD4−/− animals [6] have all been shown to be protected from IRI. Experiments in which the protected phenotype of nude mice has been reversed by adoptive transfer of CD4+, but not CD8+lymphocytes, have been published in both renal [6] and liver ischemia [11] models. Inhibition of lymphocyte chemotaxis through manipulation of the sphingosine-1-phosphate (S1P) pathway has also been used to ameliorate IRI [5], [12], [13].

Since this data suggests that effector T cells contribute to IRI, CD4+CD25+FoxP3+T regulatory cells (Treg) have become a putative therapeutic target. Treg depletion/reconstitution experiments in renal ischemia [8], [14], and pharmacotherapies designed to enhance Treg recruitment [15] have suggested a protective role for Treg, while adoptive transfer of induced Treg showed therapeutic potential in models of renal [14] and hepatic ischemia [16], [17]. In addition to experiments, which have directly examined the potential of Treg as a cellular therapy, several molecules and pathways linked with Treg activity have also been associated with IPC. IL-10 was shown to be over-expressed in renal [14] and hepatic IPC, which conversely was abolished by IL-10 neutralizing antibodies [18]. Receptor mediated purinergic signalling with adenosine has long been implicated in protection induced by IPC [19]. Degradation of interstitial ATP to adenosine is catalysed by the ectonucleotidase CD39, which is expressed by Treg and implicated in their suppressive function [20], [21]. CD39−/− animals are susceptible to hepatic ischemic injury, whereas administration of soluble CD39 attenuated hepatic injury: furthermore, CD39 was shown to be induced by IPC [22].

The potential of immunomodulation as a therapeutic strategy for hepatic IRI was supported by our previous work, in which we showed that hemeoxygenase-1 (HO-1) protected the liver from ischemic injury by modulation of antigen presenting cell (APC) differentiation to an anti-inflammatory phenotype [23]. Subsequent work by others showed that FoxP3+Treg mediated immune suppression to requires APC HO-1 [24].

Here we have used a range of approaches to study the contribution of Treg to IPC *in vivo*, and to establish whether this cell type is likely to have translational potential in the context of hepatic ischemia. We demonstrate that in our model, Treg do not modulate ischemic injury, and are unlikely to contribute to IPC.

**Figure 1:**
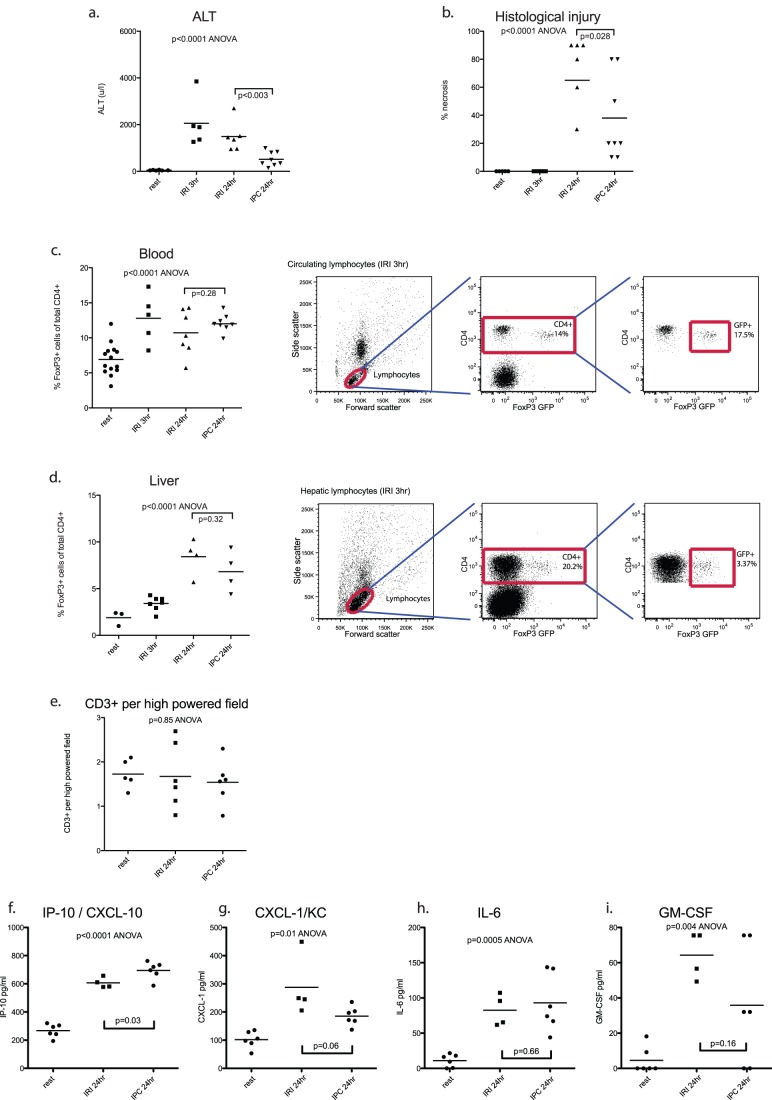
Treg mobilization during reperfusion is not enhanced by IPC. Mice (n = 5–8 per group) were subjected to ischemia reperfusion injury with or without ischemic preconditioning and killed at 3 or 24 hours. Ischemic preconditioning protected the liver from injury both in terms of ALT release [a] and histological injury score [b] measured at 24 hours. CD4+FoxP3+cells were mobilized into the circulation during reperfusion [c]. Hepatic CD4+FoxP3+cells increased over the same time period [d]. Total CD3+lymphocytes were stable throughout reperfusion [e]. FlowCytomix was used to profile circulating chemo/cytokines. Rises were detected in CXCL-10/IP-10 [f], CXCL-1/KC [g], IL-6 [h] and GMCSF [i]. Other analytes (IL-1α, IL-1β, IL-2, IFNγ, IL-17 and IL-17F, and the Treg cytokine IL-10) were not detected.

## Materials and Methods

### Ethics Statement

All procedures and animal care were approved by the University of Edinburgh Ethical Review Committee and performed in accordance with UK Home Office licensing regulations (Animal Scientific Procedures Act 1986).

**Figure 2:**
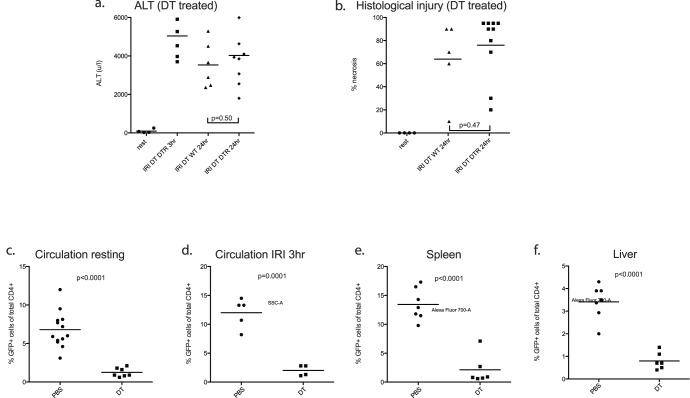
Treg depletion in the FoxP3.LuciDTR mouse does not increase susceptibility to IRI. FoxP3.LuciDTR mice or wildtype control (n = 5–10 per group) received 25 ng/g DT 24 hours prior to ischemic insult. ALT release [a] and histological injury score [b] did not differ between groups. DT treatment of DTR animals effected almost total depletion of Treg from the circulation [c,d], spleen [e] and liver [f].

### Animals, Surgical Models, and Injury Assessment

C57 BL/6J animals with insertion of a BAC containing a cassette encoding enhanced GFP (eGFP), the human DTR, and CBGr99 luciferase within exon 3 of the FoxP3 gene (FoxP3.LuciDTR) mice were made available by G. Hammerling, Heidelberg [25]. Foxp3 GFP reporter mice on the C57BL/6J background were provided by Dr. A. Rudensky, Seattle [26].

**Figure 3:**
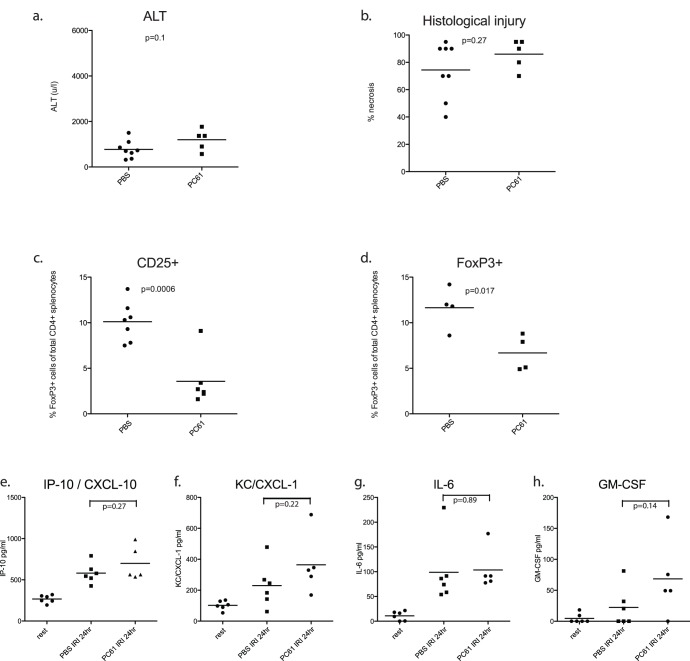
Treg antibody depletion does not increase susceptibility to IRI. Wildtype mice received the CD25 depleting antibody pc61 7 and 2 days or PBS (n = 5–8 per group) prior to ischemia reperfusion injury. Injury severity was no different between Treg intact and Treg depleted animals in terms of ALT [a] or histological injury score [b]. PC61 treatment resulted in significant depletion of CD4+CD25+[c] and CD4+FoxP3+[d] cells. FlowCytomix was used to profile circulating chemo/cytokines. No differences were detected in post-operative rises in CXCL-10/IP-10 [e], KC/CXCL-1 [f], IL-6 [g] and GM-CSF [h] between Treg intact and depleted animals. Other analytes (IL-1α, IL-1β, IL-2, IFNγ, IL-17 and IL-17F, and IL-10) were not detected.

IRI animals underwent surgical occlusion of the portal pedicle (hepatic artery and portal vein) supplying the left hepatic lobe for 50 minutes under isoflurane anaesthesia as described by previously [23], [27], [28]. IPC mice, underwent portal pedicle occlusion for 15 minutes followed by 15 minutes reperfusion prior to the 50 minute ischemic insult. Core temperature was maintained at 36°C throughout. Animals were culled at various times post-operatively. All experiments were internally controlled.

**Figure 4:**
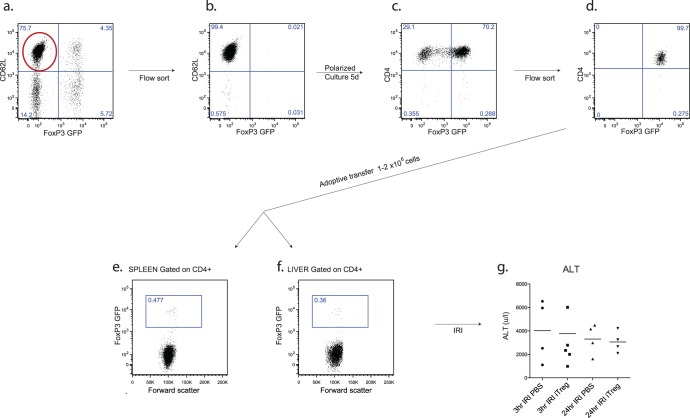
Adoptive transfer of pre-activated iTreg does not protect animals from IRI. iTreg were generated *in vitro* from sorted CD62L high, FoxP3- naïve T cells cultured in the presence of TGFβ and IL-2 for 5 days before flow sorting to maximize purity [a-d]. Successful transfer was confirmed by detection of FoxP3 GFP+cells in spleen [e] and liver [f]. No difference was detected in injury severity between iTreg supplemented and PBS treated control animals at 3 or 24 hours of reperfusion (n = 4–5 per group) [g].

Severity of ischemic injury was assessed by measurement of serum ALT utilising a commercial kit (Alpha Laboratories Ltd., Eastleigh, UK) adapted for use on a Cobas Fara centrifugal analyser (Roche Diagnostics Ltd, Welwyn Garden City, UK). Histological injury was evaluated on haematoxylin and eosin stained sections at 100x magnification on a bright field microscope. Percentage area necrosis was estimated on representative sections.

**Figure 5:**
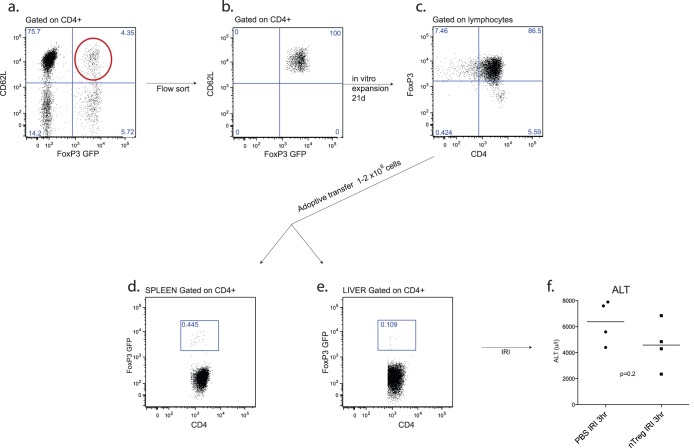
Adoptive transfer of pre-activated nTreg does not protect animals from IRI. nTreg were obtained from FoxP3 GFP mouse spleens by flow sorting followed by expansion *in vitro* for 21 days and further flow-sorting for purity [a–c]. Flow cytometry of spleen and liver confirmed successful transfer [d,e]. There was no difference in injury severity between nTreg supplemented and control animals at 3 hours of reperfusion (n = 4 per group) [f].

### Reagents

Diphtheria toxin (DT) 25 ng/g (Sigma-Aldrich, Poole, UK) was used to deplete FoxP3 Treg in FoxP3.LuciDTR animals 24 hr prior to surgery. DT treated wildtype littermates served as controls. Adequate depletion of Treg was confirmed by loss of CD4+GFP (FoxP3)+cells from tail vein blood samples prior to surgery.

Wildtype C57BL/6J animals underwent Treg depletion using PC61, a CD25 depleting antibody (Bio × Cell, New Hampshire, USA), 7 and 2 days pre-operatively. CD25+and FoxP3+depletion was confirmed by flow cytometry of splenocytes in post-operative animals.

**Figure 6:**
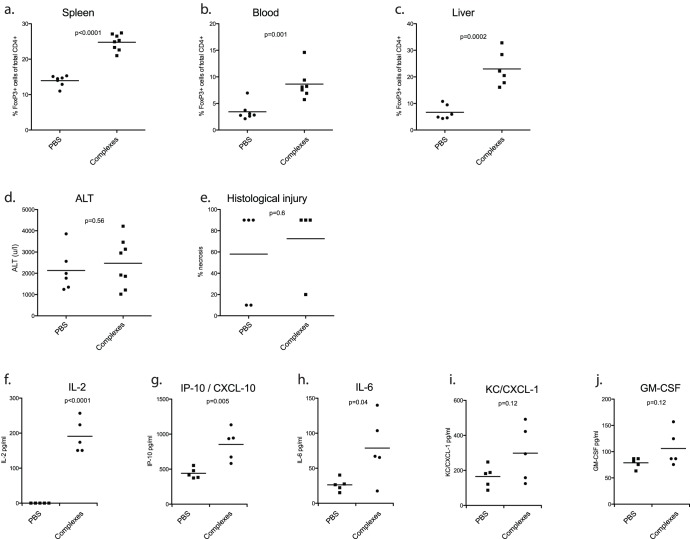
*In vivo* expansion of Treg does not protect animals from IRI. Mice received IL-2/JES6-A12 complexes or PBS for three consecutive days prior to hepatic ischemia reperfusion insults (n = 8 per group). Animals were culled at 24 hours of reperfusion, and tissues analysed for Treg frequency. There was significant expansion of splenic [a], circulating [b] and hepatic [c] Treg. Injury severity was similar between Treg expanded and control animals, in terms of ALT and histological injury [d,e]. FlowCytomix was used to profile circulating chemo/cytokines. Levels of IL-2 [f], CXCL-10/IP-10 [g] and IL-6 [h] were elevated. Rises in KC/CXCL-1 [i], and GM-CSF [j] did not differ between groups. Other analytes (IL-1α, IL-1β, IL-10, IL-17, IL-17F, IFNγ, MIP-1α) were not detected.

IL-2/anti-IL2 complexes were prepared using Garvan Institute laboratory protocols as described previously [29]. Mice received pre-incubated complexes containing 1 ug IL-2 (Peprotech, London, UK) and 5 ug anti-IL-2 Ab (clone JES6/A12, eBioscience, Hatfield, UK) on three consecutive days 72, 48, and 24 hours prior to surgery.

### Flow Cytometry

Hepatic lymphocytes were prepared from liver samples by tissue disaggregation using Miltenyi Gentlemacs (Miltenyi Biotec, UK), followed by digestion by Collagenase D (Roche, Burgess Hill, UK), and passage through a 70 µm filter. Cells were layered onto lympholyte M (Cedarlane, Ontario, Canada) for density centrifugation and separation of lymphocytes.

Splenocytes were preparared by passage through a 40 µm filter followed by red cell lysis with ammonium chloride buffer (Sigma Aldrich, Dorset, UK). Blood was collected from tail vein or cardiac puncture into EDTA buffer. Erythrocytes were lyzed with ammonium chloride buffer.

Samples containing approximately 10^6^ cells were stained as follows. Cell surface antigens were stained with antibodies to CD4+AF700 (Caltag Medsystems, Buckingham UK) and CD62L PE for 20 minutes at room temperature at dilutions of 1∶200 (CD4) and 1∶100 (others). Intracellular staining of FoxP3 or isotype control (eBioscience, Hatfield, UK), utilized a fixation/permeabilization kit (eBioscience) according to the manufacturer’s instructions. Antibodies were incubated at 1∶100 dilution for 30 minutes.

Flow cytometry was performed using a LSR Fortessa cytometer (Becton Dickinson UK), gated on lymphocytes identified by forward and side scatter characteristics.

### Immunohistochemistry and Cell Counting

Tissue samples were formalin fixed prior to paraffin embedding. Sections were stained with rabbit anti mouse CD3 (Sigma-Aldrich, Poole, Dorset, UK) at 1∶1000 dilution. Staining was detected with diaminobenzidine with haematoxylin counterstaining. Numbers of stained cells were counted on 400x fields selected at random by isotropic uniform random sampling (Image pro-plus software, Media Cybernetics Inc, CA, USA) using a Zeiss Axis A1 (Carl Zeiss Ltd, Cambridge, UK) stereology microscope.

### Measurement of Circulating Cytokines

Serum samples were analysed for IP-10, GM-CSF, IL-1α, IL-1β, IL-2, IL-6, IL-10, IL-17, IL-17F, CXCL-1/KC, MIP-1α, MIP-1β and IFNγ using a multiplex FlowCytomix kit (eBioscience) according to the manufacturer’s instructions. Data was collected using an LSR Fortessa cytometer (Becton Dickinson, UK) and analysed with Flowcytomix Pro v2.4 software (eBioscience).

### Preparation of Cells for Adoptive Transfer

iTreg were generated by Miltenyi Macs negative selection of CD4 cells from FoxP3 GFP mouse spleens (Miltenyi Biotec, UK). CD4+CD62L high GFP- naïve T cells were subsequently purified by FACS sorting. These were plated into anti-CD3/anti-CD28 coated plates in the presence of 5 ng/ml TGFβ and 100 U/ml IL-2 and cultured for 5 days [30]. iTreg were purified using FACS sorting for CD4+GFP+prior to adoptive transfer of 1–2 million cells 24 hours prior to ischemic insult. Successful engraftment of donor cells was confirmed by flow cytometry of recipient blood, liver and spleen.

nTreg were obtained by Miltenyi Macs negative selection of CD4 cells from FoxP3 GFP mouse spleens, followed by FACS sorting. FoxP3 expression was previously shown to be more stable in CD4+CD62L high FoxP3+cells [31], therefore this subgroup was selected for ex-vivo expansion. Around 2–3×10^5^ nTreg were recovered from each donor mouse, which underwent ex vivo expansion in the presence of Dynal CD3/CD28 activation beads (Life Technologies, Paisley, UK) and high dose (2000U/ml) IL-2 in order to produce around 10–20 fold expansion at 21 days.

### Statistical Analysis

Data were collected in Microsoft Excel for Mac and analysed using Prism version 5 (Graphpad, San Diego, USA). Data are presented as mean+/− standard error. Comparison of means was with 2 tailed t test or ANOVA as appropriate.

## Results

### Treg Mobilization during Reperfusion

IPC protected the liver from injury both in terms of ALT release and histological injury score measured at 24 hours [Figure 1a,b]. Early reperfusion was accompanied by a significant mobilization of FoxP3+T regs into the circulation, rising to between 10 and 15% of circulating CD4+cells at 3 hours, and remaining elevated at later time points [Figure 1c]. In turn, the liver accumulated T regs, rising to 5–10% of hepatic CD4+cells at 24 hours [Figure 1d]. Total numbers of CD3+lymphocytes detected on immunohistochemistry was stable throughout reperfusion [Figure 1e].

Importantly, despite the significant protection from injury offered by IPC, numbers of Treg in the circulation and enrichment within the liver was similar between IRI and IPC mice [Figure 1c, d]. Thus enrichment with Treg was not associated with a protected phenotype.

To further evaluate whether IPC might be associated with Treg activity, serum chemokine and cytokine profiles were compared between animals subject to IRI and IPC. Cytokines IL-1α, IL-1β, IL-2, IFNγ, IL-17 and IL-17F, and IL-10 were not detected in the serum of either IRI or IPC animals. There were rises in CXCL-10/IP-10 [Figure 1f], KC/CXCL-1 [Figure 1g], IL-6 [Figure 1h] and GM-CSF [Figure 1i].

Apart from a small difference in levels of serum CXCL-10/IP-10, and a trend towards lower KC/CXCL-1 in IPC mice (p = 0.06), chemokine and cytokine profiles did not significantly differ between IRI and IPC groups [Figure 1f, g, h, i]. Specifically, IPC was not associated with significant reductions in pro-inflammatory cytokines or an excess of IL-10, which may have implicated a role for Treg.

### Effect of T Regulatory Cell Depletion

If Treg were responsible for modulating ischemic injury, then Treg deficient animals would be more susceptible to injury. To test this hypothesis, we depleted animals of Treg using two complementary approaches.

Foxp3.LuciDTR mice or wildtype littermates received diphtheria toxin 25 ng/g 24 hours prior to IRI. Loss of CD4+FoxP3+cells, from blood [Figure 2c,d], spleen [Figure 2e], and liver [Figure 2f] was profound. Despite this, injury severity was equal between Treg depleted Foxp3.LuciDTR and DT treated Treg intact wildtype littermate control animals at 24 hours of reperfusion [Figure 2a, b].

Since DT treatment is toxic, and exacerbated IRI in these experiments, we verified the absence of Treg mediated protection using antibody depletion. Mice received two doses of the monoclonal anti CD25 antibody PC61 or PBS control 7 days and 2 days prior to surgery, as described previously by our laboratory [32]. During protocol optimization, this regimen reduced numbers of CD25+cells to around one third of their original number, and approximately halved the proportion of splenic FoxP3+CD4+cells [Figure 3c,d]. PC61 Treg depleted mice were no more susceptible to IRI in terms of ALT or histological injury score than Treg intact animals [Figure 3a,b].

FlowCytomix was used to measure cytokine and chemokine levels in serum from animals undergoing antibody mediated Treg depletion prior to ischemic insult. No differences were detected between Treg depleted and vehicle treated animals [Figure 3e, f, g, h], specifically, the relative absence of Treg did not result in elevated levels of circulating pro-inflammatory cytokines at 24 hours of reperfusion. Cytokines IL-1α, IL-1β, IL-2, IFNγ, IL-17 and IL-17F, and IL-10 were not detected in the serum of Treg intact or Treg depleted animals at all [data not shown]. Post-operative rises in CXCL-10/IP-10 [Figure 3e], KC/CXCL-1 [Figure 3f], IL-6 [Figure 3g] and GM-CSF [Figure 3h] did not differ between groups. Of note, secretion of pro-inflammatory chemokines and cytokines was not exacerbated in Treg depleted animals.

The lack of increased susceptibility of Treg depleted mice to ischemic injury suggests that Treg do not have a physiological role in modulating injury, and are unlikely to be responsible for the protected phenotype observed in IPC.

### Testing the Therapeutic Potential of Transferred or Expanded Pre-activated Treg

If Treg were capable of ameliorating ischemic injury, expansion of Treg numbers might be protective. To test this hypothesis we supplemented animals with additional activated Treg using techniques shown to protect animals from other inflammatory pathologies within our laboratory.

Activated FoxP3 GFP iTreg were generated in vitro using techniques previously published by our laboratory [30], and purified to ≈99% by FACS sorting, before adoptive transfer of 1–2 million cells [Figure 4a, b, c, d]. 24 hours later, animals were subjected to hepatic ischemic insult. Successful engraftment of donor cells was confirmed by flow cytometry of recipient spleen [Figure 4e] and liver [Figure 4f]. Cells were prepared and transferred using procedures and reagents shown to confer protection in other models in reports from our laboratory [30]. No difference in injury severity was observed between recipients of adoptively transferred iTreg and control animals [Figure 4g].

Since previous reports have shown iTreg to produce IFNγ when stimulated [30], and FoxP3 GFP reporter iTreg have been shown to exhibit reduced stability of FoxP3 expression and reduced suppressive ability [33], experiments were repeated using adoptively transferred nTreg.

1–2 million cells were transferred into recipient mice prior to ischemic injury [Figure 5a, b, c]. Despite engraftment of nTreg [Figure 5d,e], mice were not protected from ischemic injury in terms of ALT at 3 hours of reperfusion [Figure 5f].

A possible explanation of the failure of adoptive transfer experiments to protect animals from ischemic injury was that insufficient Treg had been transferred to ameliorate injury. IL-2/anti-IL-2 complexes have been shown to induce Treg expansion, protecting experimental models from airway inflammation [34], experimental autoimmune encephalomyelitis (EAE) [29], and conferring long term tolerance to pancreatic allografts [29]. Prior to ischemia reperfusion insults, animals received three consecutive daily injections of IL-2/anti-IL-2 complexes resulting in substantial Treg expansion in spleen [Figure 6a], blood [Figure 6b] and liver [Figure 6c].

Despite a two to threefold expansion in Treg, including in the liver, animals were not protected from injury in terms of ALT release or histological injury score at 24 hours of reperfusion [Figure 6d,e].

Serum cytokine/chemokine profiles showed increased levels of IL-2 in keeping with exogenous administration [Figure 6f]. Interestingly, CXCL-10/IP-10 was elevated in complex treated animals compared with PBS treated controls [Figure 6g], as was IL-6 [Figure 6h]. KC/CXCL-1 [Figure 6i], and GM-CSF [Figure 6j] did not differ between groups. Other analytes (IL-1α, IL-1β, IL-10, IL-17, IL-17F, IFNγ, MIP-1α) were not detected in either group.

## Discussion

These experiments, which employed a range of strategies of Treg supplementation/expansion previously shown to ameliorate other inflammatory disorders, demonstrate that Treg are unlikely to represent a useful therapeutic target in amelioration of hepatic IRI.

We have shown a rapid mobilization of Treg into the circulation and accumulation in the liver during the first 24 hours of reperfusion. This mobilization and accumulation was not exaggerated by IPC, and did not inversely correlate with severity of ischemic injury, as would have been predicted had they had a significant role in injury modulation.

Profound depletion of Treg in the FoxP3 LuciDTR mouse did not affect injury compared with appropriate DT treated control animals. Antibody depletion of CD25+cells (which led to partial loss of FoxP3+Treg) also did not adversely affect outcome of ischemia reperfusion insults. If Treg were protective, injury would have been more severe in Treg deficient animals than Treg intact controls.

Conversely, if Treg were implicated in IPC, supplementation or expansion of these cells would be expected to recapitulate the protected preconditioned phenotype. We employed three putative therapeutic strategies of either Treg transfer or expansion, in attempts to ameliorate injury. Pre-activated iTreg have previously been used in our laboratory to ameliorate experimental autoimmune encephalomyelitis (EAE) [30]. In our ischemia model, we were unable to demonstrate protection. We hypothesized that a protective effect might be undermined by potentially pathological IFNγ production by iTreg [30]. We also noted recent data demonstrating impaired suppressive capability and FoxP3 stability in FoxP3 GFP iTreg [33]. Thus, we undertook further experiments using transferred ex vivo expanded nTreg [31] which again did not protect the liver from ischemic injury.

A limitation of adoptive transfer experiments is their inability to significantly expand circulating or tissue Treg numbers. We transferred similar numbers of iTreg and nTreg to those shown to protect animals from other inflammatory disorders, and transferred cells which were pre-activated, increasing their likely suppressive potency. Nonetheless, 1–2 million Treg remains a small proportion of the total number within the host mouse, and donor cells were detected in the liver and spleen at around 0.5% CD4+cells.

To address this issue, we utilised IL-2/anti IL-2 complexes, which have been shown to ameliorate airway inflammation [34], and EAE, as well as inducing long term tolerance to transplanted pancreatic islets [29]. In common with previously published reports, this technique produced major Treg expansion in the spleen, circulation and liver. Despite 25% of hepatic CD4+cells being FoxP3+, the ischemic preconditioned phenotype was not recapitulated by *in vivo* Treg expansion.

It would be possible to ascribe a lack of protective effect in Treg transferred or expanded animals to those cells’ polyclonal TCR repertoire. Previous work using transgenic TCR restricted models highlighted that Treg recognising a disease-relevant autoantigen are more effective than polyclonal Treg in prevention of EAE [31]. In contrast, ischemic injury is likely to result in unveiling of a wide range of antigens as the contents of lysing cells are “spilt” into the interstitium. As such, TCR restricted models are not only unavailable, but they are also unlikely to be therapeutically relevant in the context of IRI.

In these experiments, we have used a number of complementary approaches to establish whether Treg have a role in modulating hepatic IRI, and have found no data to support this hypothesis. Other published Treg depletion experiments have had mixed results using the PC61 anti-CD25 antibody, with some showing increased susceptibility [8], [14], [35], and others unable to demonstrate an effect [15], [36] in IRI models. Results of Treg supplementation experiments from other laboratories contrast with our own data. Transfer of iTreg cells into mice [16] and rats [17] have been shown by others to be protective against hepatic IRI. RAG1 knockout mice, which underwent reconstitution with FoxP3−/− lymphocytes were highly vulnerable to ischemic injury compared with recipients of wildtype lymphocytes [8]. In a study of late phase preconditioning (in which the preconditioning insult occurred 7 days prior to index ischemia), the same group showed that Treg accumulated during resolution of injury, and that these partially reduced the severity of subsequent ischemic insults [14].

The work presented here demonstrates an important negative finding, contrary to our original hypothesis, that Treg are unlikely to be responsible for IPC. Clearly this cell type represents an important therapeutic target for the treatment of a wide range of immune mediated disorders, but it is unlikely to be of clinical value in the treatment of ischemia reperfusion injury.

## References

[1] Devey LR, Friend PJ, Forsythe JL, Mumford LL, Wigmore SJ (2007) The use of marginal heart beating donor livers for transplantation in the United kingdom. Transplantation 84: 70–74. 17627240 10.1097/01.tp.0000268072.04260.69

[2] Clavien PA, Yadav S, Sindram D, Bentley RC (2000) Protective effects of ischemic preconditioning for liver resection performed under inflow occlusion in humans. Annals of surgery 232: 155–162. 10903590 10.1097/00000658-200008000-00001 PMC1421123

[3] Petrowsky H, McCormack L, Trujillo M, Selzner M, Jochum W, et al.. (2006) A prospective, randomized, controlled trial comparing intermittent portal triad clamping versus ischemic preconditioning with continuous clamping for major liver resection. Annals of surgery 244: 921–928; discussion 928–930. 10.1097/01.sla.0000246834.07130.5d PMC1856627 17122617

[4] Caldwell CC, Okaya T, Martignoni A, Husted T, Schuster R, et al (2005) Divergent functions of CD4+T lymphocytes in acute liver inflammation and injury after ischemia-reperfusion. American journal of physiology Gastrointestinal and liver physiology 289: G969–976. 16002566 10.1152/ajpgi.00223.2005

[5] Lai LW, Yong KC, Igarashi S, Lien YH (2007) A sphingosine-1-phosphate type 1 receptor agonist inhibits the early T-cell transient following renal ischemia-reperfusion injury. Kidney international 71: 1223–1231. 17377506 10.1038/sj.ki.5002203

[6] Burne MJ, Daniels F, El Ghandour A, Mauiyyedi S, Colvin RB, et al (2001) Identification of the CD4(+) T cell as a major pathogenic factor in ischemic acute renal failure. The Journal of clinical investigation 108: 1283–1290. 11696572 10.1172/JCI12080 PMC209434

[7] Burne-Taney MJ, Liu M, Baldwin WM, Racusen L, Rabb H (2006) Decreased capacity of immune cells to cause tissue injury mediates kidney ischemic preconditioning. Journal of immunology 176: 7015–7020. 10.4049/jimmunol.176.11.7015 16709863

[8] Kinsey GR, Sharma R, Huang L, Li L, Vergis AL, et al (2009) Regulatory T cells suppress innate immunity in kidney ischemia-reperfusion injury. Journal of the American Society of Nephrology : JASN 20: 1744–1753. 19497969 10.1681/ASN.2008111160 PMC2723989

[9] Horie Y, Wolf R, Chervenak RP, Jennings SR, Granger DN (1999) T-lymphocytes contribute to hepatic leukostasis and hypoxic stress induced by gut ischemia-reperfusion. Microcirculation 6: 267–280. 10654278

[10] Savransky V, Molls RR, Burne-Taney M, Chien CC, Racusen L, et al (2006) Role of the T-cell receptor in kidney ischemia-reperfusion injury. Kidney international 69: 233–238. 16408111 10.1038/sj.ki.5000038

[11] Zwacka RM, Zhang Y, Halldorson J, Schlossberg H, Dudus L, et al (1997) CD4(+) T-lymphocytes mediate ischemia/reperfusion-induced inflammatory responses in mouse liver. The Journal of clinical investigation 100: 279–289. 9218504 10.1172/JCI119533 PMC508190

[12] Anselmo DM, Amersi FF, Shen XD, Gao F, Katori M, et al (2002) FTY720 pretreatment reduces warm hepatic ischemia reperfusion injury through inhibition of T-lymphocyte infiltration. American journal of transplantation : official journal of the American Society of Transplantation and the American Society of Transplant Surgeons 2: 843–849. 10.1034/j.1600-6143.2002.20906.x 12392290

[13] Lien YH, Yong KC, Cho C, Igarashi S, Lai LW (2006) S1P(1)-selective agonist, SEW2871, ameliorates ischemic acute renal failure. Kidney international 69: 1601–1608. 16572108 10.1038/sj.ki.5000360

[14] Kinsey GR, Huang L, Vergis AL, Li L, Okusa MD (2010) Regulatory T cells contribute to the protective effect of ischemic preconditioning in the kidney. Kidney international 77: 771–780. 20164824 10.1038/ki.2010.12 PMC2912287

[15] Lai LW, Yong KC, Lien YH (2012) Pharmacologic recruitment of regulatory T cells as a therapy for ischemic acute kidney injury. Kidney international 81: 983–992. 22189844 10.1038/ki.2011.412 PMC3340526

[16] Feng M, Wang Q, Zhang F, Lu L (2012) Ex vivo induced regulatory T cells regulate inflammatory response of Kupffer cells by TGF-beta and attenuate liver ischemia reperfusion injury. International immunopharmacology 12: 189–196. 22155100 10.1016/j.intimp.2011.11.010

[17] Lu L, Li G, Rao J, Pu L, Yu Y, et al (2009) In vitro induced CD4(+)CD25(+)Foxp3(+) Tregs attenuate hepatic ischemia-reperfusion injury. International immunopharmacology 9: 549–552. 19539564 10.1016/j.intimp.2009.01.020

[18] Serafin A, Rosello-Catafau J, Prats N, Gelpi E, Rodes J, et al (2004) Ischemic preconditioning affects interleukin release in fatty livers of rats undergoing ischemia/reperfusion. Hepatology 39: 688–698. 14999687 10.1002/hep.20089

[19] Linden J (2001) Molecular approach to adenosine receptors: receptor-mediated mechanisms of tissue protection. Annual review of pharmacology and toxicology 41: 775–787. 10.1146/annurev.pharmtox.41.1.775 11264476

[20] Deaglio S, Dwyer KM, Gao W, Friedman D, Usheva A, et al (2007) Adenosine generation catalyzed by CD39 and CD73 expressed on regulatory T cells mediates immune suppression. The Journal of experimental medicine 204: 1257–1265. 17502665 10.1084/jem.20062512 PMC2118603

[21] Fletcher JM, Lonergan R, Costelloe L, Kinsella K, Moran B, et al (2009) CD39+Foxp3+regulatory T Cells suppress pathogenic Th17 cells and are impaired in multiple sclerosis. Journal of immunology 183: 7602–7610. 10.4049/jimmunol.0901881 19917691

[22] Hart ML, Gorzolla IC, Schittenhelm J, Robson SC, Eltzschig HK (2010) SP1-dependent induction of CD39 facilitates hepatic ischemic preconditioning. Journal of immunology 184: 4017–4024. 10.4049/jimmunol.0901851 PMC2846294 20207994

[23] Devey L, Ferenbach D, Mohr E, Sangster K, Bellamy CO, et al (2009) Tissue-resident macrophages protect the liver from ischemia reperfusion injury via a heme oxygenase-1-dependent mechanism. Mol Ther 17: 65–72. 19002167 10.1038/mt.2008.237 PMC2834991

[24] George JF, Braun A, Brusko TM, Joseph R, Bolisetty S, et al (2008) Suppression by CD4+CD25+regulatory T cells is dependent on expression of heme oxygenase-1 in antigen-presenting cells. The American journal of pathology 173: 154–160. 18511516 10.2353/ajpath.2008.070963 PMC2438293

[25] Suffner J, Hochweller K, Kuhnle MC, Li X, Kroczek RA, et al (2010) Dendritic cells support homeostatic expansion of Foxp3+regulatory T cells in Foxp3.LuciDTR mice. Journal of immunology 184: 1810–1820. 10.4049/jimmunol.0902420 20083650

[26] Fontenot JD, Rasmussen JP, Williams LM, Dooley JL, Farr AG, et al (2005) Regulatory T cell lineage specification by the forkhead transcription factor foxp3. Immunity 22: 329–341. 15780990 10.1016/j.immuni.2005.01.016

[27] Devey L, Festing MF, Wigmore SJ (2008) Effect of temperature control upon a mouse model of partial hepatic ischaemia/reperfusion injury. Lab Anim 42: 12–18. 18348762 10.1258/la.2007.06009e

[28] Devey L, Mohr E, Bellamy C, Simpson K, Henderson N, et al (2009) c-Jun terminal kinase-2 gene deleted mice overexpress hemeoxygenase-1 and are protected from hepatic ischemia reperfusion injury. Transplantation 88: 308–316. 19667931 10.1097/TP.0b013e3181ae3067

[29] Webster KE, Walters S, Kohler RE, Mrkvan T, Boyman O, et al (2009) In vivo expansion of T reg cells with IL-2-mAb complexes: induction of resistance to EAE and long-term acceptance of islet allografts without immunosuppression. The Journal of experimental medicine 206: 751–760. 19332874 10.1084/jem.20082824 PMC2715127

[30] O’Connor RA, Leech MD, Suffner J, Hammerling GJ, Anderton SM (2010) Myelin-reactive, TGF-beta-induced regulatory T cells can be programmed to develop Th1-like effector function but remain less proinflammatory than myelin-reactive Th1 effectors and can suppress pathogenic T cell clonal expansion in vivo. Journal of immunology 185: 7235–7243. 10.4049/jimmunol.1001551 21084662

[31] Stephens LA, Malpass KH, Anderton SM (2009) Curing CNS autoimmune disease with myelin-reactive Foxp3+Treg. European journal of immunology 39: 1108–1117. 19350586 10.1002/eji.200839073

[32] Leech MD, Benson RA, De Vries A, Fitch PM, Howie SE (2007) Resolution of Der p1-induced allergic airway inflammation is dependent on CD4+CD25+Foxp3+regulatory cells. Journal of immunology 179: 7050–7058. 10.4049/jimmunol.179.10.7050 17982096

[33] Bettini ML, Pan F, Bettini M, Finkelstein D, Rehg JE, et al (2012) Loss of epigenetic modification driven by the Foxp3 transcription factor leads to regulatory T cell insufficiency. Immunity 36: 717–730. 22579476 10.1016/j.immuni.2012.03.020 PMC3361541

[34] Wilson MS, Pesce JT, Ramalingam TR, Thompson RW, Cheever A, et al (2008) Suppression of murine allergic airway disease by IL-2:anti-IL-2 monoclonal antibody-induced regulatory T cells. Journal of immunology 181: 6942–6954. 10.4049/jimmunol.181.10.6942 PMC2706157 18981114

[35] Monteiro RM, Camara NO, Rodrigues MM, Tzelepis F, Damiao MJ, et al (2009) A role for regulatory T cells in renal acute kidney injury. Transplant immunology 21: 50–55. 19233269 10.1016/j.trim.2009.02.003

[36] Kuboki S, Sakai N, Tschop J, Edwards MJ, Lentsch AB, et al (2009) Distinct contributions of CD4+T cell subsets in hepatic ischemia/reperfusion injury. American journal of physiology Gastrointestinal and liver physiology 296: G1054–1059. 19264952 10.1152/ajpgi.90464.2008 PMC2696215

